# The Effect of Environmental Enrichment on Glutathione-Mediated Xenobiotic Metabolism and Antioxidation in Normal Adult Mice

**DOI:** 10.3389/fneur.2018.00425

**Published:** 2018-07-04

**Authors:** Jung Hwa Seo, Soonil Pyo, Yoon-Kyum Shin, Bae-Geun Nam, Jeong Won Kang, Kwang Pyo Kim, Hoo Young Lee, Sung-Rae Cho

**Affiliations:** ^1^Department and Research Institute of Rehabilitation Medicine, Yonsei University College of Medicine, Seoul, South Korea; ^2^Brain Korea 21 PLUS Project for Medical Science, Yonsei University College of Medicine, Seoul, South Korea; ^3^Graduate Program of NanoScience and Technology, Yonsei University, Seoul, South Korea; ^4^Department of Applied Chemistry, Kyung Hee University, Yongin, South Korea; ^5^Precision Medicine Branch, Research Institute, National Cancer Center, Goyang, South Korea; ^6^Department of Medicine, The Graduate School of Yonsei University, Seoul, South Korea; ^7^TBI Rehabilitation Center, National Traffic Injury Rehabilitation Hospital, Yangpyeong, South Korea; ^8^Department of Rehabilitation Medicine, School of Medicine, Seoul St. Mary's Hospital, The Catholic University of Korea, Seoul, South Korea; ^9^Yonsei Stem Cell Research Center, Avison Biomedical Research Center, Seoul, South Korea; ^10^Rehabilitation Institute of Neuromuscular Disease, Yonsei University College of Medicine, Seoul, South Korea

**Keywords:** environmental enrichment, olfactory bulb, metabolizing enzymes, antioxidant, detoxification, glutathione

## Abstract

Olfactory bulb (OB) plays an important role in protecting against harmful substances via the secretion of antioxidant and detoxifying enzymes. Environmental enrichment (EE) is a common rehabilitation method and known to have beneficial effects in the central nervous system. However, the effects of EE in the OB still remain unclear. At 6 weeks of age, CD-1® (ICR) mice were assigned to standard cages or EE cages. After 2 months, we performed proteomic analysis. Forty-four up-regulated proteins were identified in EE mice compared to the control mice. Gene Ontology analysis and Kyoto Encyclopedia of Genes and Genomes Pathway demonstrated that the upregulated proteins were mainly involved in metabolic pathways against xenobiotics. Among those upregulated proteins, 9 proteins, which participate in phase I or II of the xenobiotic metabolizing process and are known to be responsible for ROS detoxification, were validated by qRT-PCR. To explore the effect of ROS detoxification mediated by EE, glutathione activity was measured by an ELISA assay. The ratio of reduced glutathione to oxidized glutathione was significantly increased in EE mice. Based on a linear regression analysis, GSTM2 and UGT2A1 were found to be the most influential genes in ROS detoxification. For further analysis of neuroprotection, the level of iNOS and the ratio of Bax to Bcl-2 were significantly decreased in EE mice. While TUNEL^+^ cells were significantly decreased, Ki67^+^ cells were significantly increased in EE mice, implicating that EE creates an optimal state for xenobiotic metabolism and antioxidant activity. Taken together, our results suggested that EE protects olfactory layers via the upregulation of glutathione-related antioxidant and xenobiotic metabolizing enzymes, eventually lowering ROS-mediated inflammation and apoptosis and increasing neurogenesis. This study may provide an opportunity for a better understanding of the beneficial effects of EE in the OB.

## Introduction

The olfactory bulb (OB) is a region of brain that plays a critical role in health and pathological behaviors such as the detection of hazards in the environment, food selection, olfactory memory formation, mating, and maintenance of mood (1–4). Tight communication between the OB and other olfactory systems can transfer xenobiotics to the brain by the active axonal transport of olfactory neurons, bypassing the blood brain barrier (5). To maintain cellular integrity and homeostasis of the brain from environmental chemicals, it is necessary to have active xenobiotic metabolism during the olfactory transport (6).

Xenobiotic metabolism is a metabolism pathway that modifies the chemical structure of foreign, either exogenous or endogenous, substances so that maintain homeostasis and cellular integrity. Xenobiotic metabolism process includes three steps; Phase I modifies xenobiotics via hydrolysis, oxidation, and reduction and thereby produces the active metabolites. Phase II further conjugates the phase I product with transferase enzymes to yield hydrophilic products. Lastly, Phase III excretes the final modified product out of the cells via various transporters (6). Namely, metabolizing enzymes such as the cytochrome P450 (CYPs) family, the UDP-glucuronosyltransferases (UGT) family, glutathione-transferases (GST) family and the aldehyde dehydrogenase (ADH) family are involved in the detoxification process (6, 7). Especially, the phase II enzymes such as UGT and GST family plays an important role in determining the rate of xenobiotic metabolism and ROS detoxification (8).

Previous studies have shown olfactory bulbectomy in rodents leads to cortical and hippocampal cell death (9, 10), suggesting that the OB is related to other brain regions. Moreover, the OB is a special nervous structure that changes its neural cells over lifetime. Several studies have shown that newly generated cells in the subventricular zone (SVZ) of adult mouse migrate into the OB through the rostral migratory stream (RMS), forming functional granule cells in the OB and constituting the network of the OB to other brain regions (11, 12). Since the OB constantly interacts with external environment and encounters harmful air-borne chemicals, the above ongoing process is important for proper OB function (13).

Environmental enrichment (EE) is a rehabilitation strategy for rodents and the enhancement of the brain by surroundings that include various objects such as running wheels, novel toys and allow large social interactions to experience the effects of a complex combination of physical, cognitive, and social stimulation in the brain (14–16). In many studies, EE has been implicated in improvement of the brain functions such as neuroprotection and neurogenesis in an animal model of aging (17–20), ischemic brain injury (14), and neurodegenerative diseases (14, 21). These diseases and brain injuries, accompanied with an increase in oxidative stress and olfactory impairment, eventually lead to a clinically significant problem (22, 23). However, this event can be reversed by exercise via the increased expression of antioxidant enzymes (17, 24).

Previous studies have investigated the effect of EE, odor enrichment, and xenobiotic inhalant on the OB of normal mice (25–27). Allowing xenobiotic inhalants or various odorants in a timely fashion, it could induce changes in gene expressions and odor perception (25, 26). Several studies have reported the beneficial effect of EE on the OB (25, 27). In contrast to the previous studies, our study focuses on the effect of EE on the OB of normal mice without utilizing any inhalant or timely-exposure odorants.

The aim of this study was to determine whether there are alterations in physiological responses after long-term exposure to EE in the intact OB of normal mice. Here, we showed the expression of metabolizing enzymes, which were predominated in the OB of EE mice. Moreover, we found that EE enhances neurogenesis and ROS detoxification, resulting in the reduction of inflammation and apoptotic process. These results imply that EE has beneficial effects on the OB through setting an optimal status for antioxidant and xenobiotic metabolism.

## Materials and methods

### Experimental procedures

#### Animals and housing conditions

**Figure 1:**
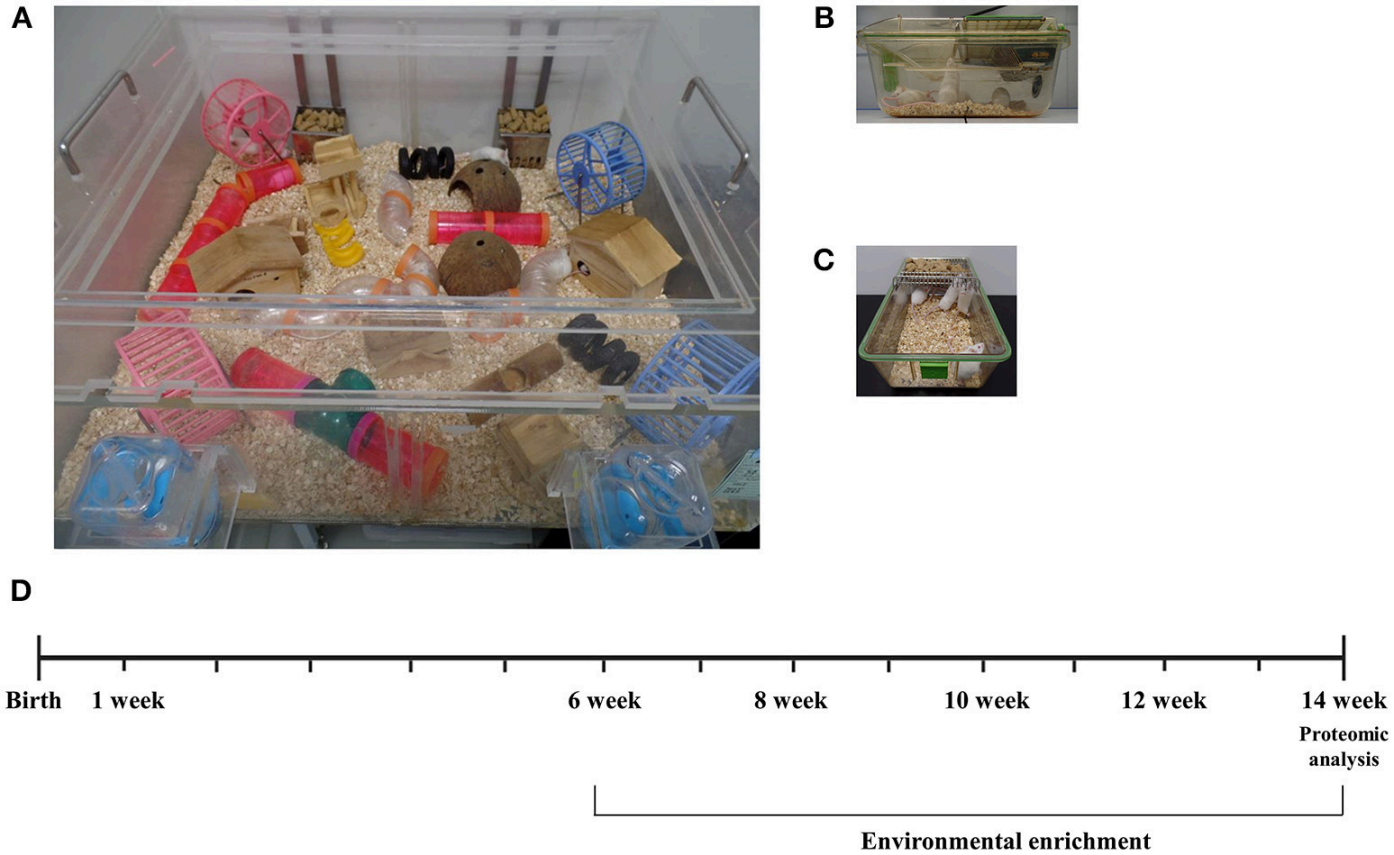
Experimental design for environmental enrichment. **(A)** Mice in an enriched environment accessed freely not only to water and food but also to tunnels, shelters, toys, running wheels, and the large space of social interaction. **(B,C)** Mice in a standard cage freely accessed to water and food. **(D)** A schematic time schedule for the experiment. Mice of 6 weeks of age were randomly assigned and reared in either a control cage or EE cage for 2 months. After the housing conditions, brain tissues were harvested for proteomic analysis to see differentially expressed proteins. Long-term exposure to EE during adulthood is known to induce the beneficial effects on brain health with cellular and molecular alterations.

At 6 weeks of age, a total of CD-1® (ICR) mice were assigned randomly to either environmental enrichment (EE) or standard cage (SC) group. For 2 months, EE mice were housed in a huge cage (86 × 76 × 31 cm^3^) containing novel objects, such as tunnels, shelters, toys, and running wheels for voluntary exercise, and allowing for large social interaction (12–15 mice/cage) (Figure 1A) while control mice were housed in standard cages (27 × 22.5 × 14 cm^3^) without any novel objects (4–5 mice/cage) (Figures 1B,C) (14). All animals were housed in a facility accredited by the Association for Assessment and Accreditation of Laboratory Animal Care (AAALAC) and were given *ad libitum* access to food and water under alternating 12-h light/dark cycles. Used mice of this study were all female mice and purchased from the Jackson Laboratory.

#### Ethic statement

All procedures were reviewed and approved by the Animal Care and Use Committee of the Yonsei University College of Medicine (Identification code: 2014-0125-3). All procedures were in accordance with the guidelines of the National Institutes of Health's Guide for the Care and Use of Laboratory Animals. These regulations, notifications, and guidelines originated and were modified from the Animal Protection Law (2008), the Laboratory Animal Act (2008), and the Eighth Edition of the Guide for the Care and Use of Laboratory Animals (NRC 2011). They were sacrificed at 8 weeks after the housing conditions under ketamine (100 mg/kg) and xylazine (10 mg/kg) anesthesia by intraperitoneal injection. Thereafter, they were given an intracardial perfusion of 4% paraformaldehyde, and the brain tissues were harvested for histological assessments. All efforts were made to minimize animal suffering.

#### Experimental grouping

In this study, a total of 28 CD-1® (ICR) female mice at 6 weeks of age were randomly housed either in environmental enrichment (*N* = 14) or standard cages (*N* = 14). Among the subjects, 3 mice per group were recruited for the proteomics analysis after housing for 2 months (*N* = 6). To validate the results of proteomics and measure iNOS levels, 4 mice per group were further recruited at 8 weeks post-conditions (*N* = 8). Moreover, 3 mice per group were recruited for an ELISA assay to measure glutathione activity and apoptosis process (*N* = 6). Finally, 4 mice per group were further recruited for histological assessments (*N* = 8). A schematic timeline for this study is described in Figure 1D.

### Proteomic analysis

#### Protein sample preparation

Olfactory bulb of mouse brain tissues was homogenized in 200 μl of cold RIPA buffer (50 mM Tris-HCl, pH 7.5, 1% Triton X-100, 150 mM NaCl, 0.1% sodium dodecyl sulfate (SDS), 1% sodium deoxycholate) with a protease inhibitor cocktail (Sigma-Aldrich, St. Louis, MO, USA) in the ice. The tissue lysates were centrifugated for 20 min at 13,000 g at 4°C. The supernatant containing the protein extracts were stored at −80°C until use. The protein concentration of the supernatant was determined using Bradford assay, according to the manufacturer's instructions.

#### In-solution digestion

Protein samples were digested by in-solution method after quantified using a bicinchoninic acid (BCA) assay kit (Thermo Scientific, Foster City, CA, USA). Briefly, samples were resolved in a digestion solution of 6 M urea (Sigma-Aldrich) and 50 mM tris-HCl in water. Protein reduction was performed with 10 mM DTT (Sigma-Aldrich) for 1 h at 37°C. Additionally, 55 mM iodoacetamide (Sigma-Aldrich) were treated for 30 min at room temperature in darkness for alkylation of cysteine residues. The resulting samples were digested by sequencing-grade modified trypsin (Promega, Madison, WI, USA) overnight at 37°C. The digested peptides were desalted using C18 Spin-Column (Thermo Scientific).

#### TMT labeling and OFF-GEL fractionation

For TMT labeling, digested peptides were resuspended in 200 mM triethyl ammonium bicarbonate (TEAB, Sigma-Aldrich). TMT™ Isobaric Mass Tagging reagent (Thermo Scientific) were dissolved in anhydrous acetonitrile and added to each sample. After incubation for 1 h at room temperature, the reaction was quenched with 5% hydroxylamine. The labeled samples were pooled into one tubes and separated with an Agilent 3100 OFF-GEL fractionator system (Agilent Technologies, Santa Clara, CA, USA) according to manufacturer's instructions. Finally, resulting samples were desalted using C18 Spin-Column (Thermo Scietific).

#### LC-MS analysis and data acquisition

Prepared samples were analyzed using Q Exactive mass spectrometer (Thermo Finnigan, San Jose, CA, USA) coupled with EASY-nLC 1000 (Thermo Finnigan). The tryptic peptides were loaded into a trap column (3 μm sized C18 resin into 75 μm × 2 cm) and separated by the analytical column (3 μm sized C18 resin into 75 μm × 50 cm) at a flow rate of 300 nL/min. The separated peptides were eluted by solvent A (0.1% formic acid in water) and solvent B (0.1% formic acid in acetonitrile) with a linear gradient for 60 min. Each MS scan was followed by 8 MS/MS scans of the most abundant peak to the eighth-most abundant peak of the MS scan in data-dependent method with dynamic exclusion options (repeat duration was 30 s and repeat count was set to 1). The normalized collision energy for MS/MS was 27%. To identify peptides, TurboSEQUEST (Thermo Electron, San Jose, CA, USA) was used to search MS/MS spectra against the Uniprot database for forward sequences (17098 *Mus musculus* protein sequence entries as of July 15, 2014). The mass tolerance was set to 10 ppm for precursor ions, 0.8 Da for fragment ions. Carbamidomethylation of cysteine (+57.0215 Da) and oxidation of methionine (+15.9949 Da) were set as the static modifications and variable modifications, respectively. Scaffold Q+S (version 4.3.4, Proteome Software Inc., OR, USA) was used to compute probability of proteins and peptides (28). Search results were validated using the protein and peptide cut-off probabilities with >99% and 95% probability, respectively. Spectral counts were calculated by Scaffold software after normalization by total spectral counts of each MS run for an estimate of expression fold change between the experimental groups (29). An absorbance spectra peak for each interested protein is provided in Figure S1.

#### Bioinformatics and pathway analysis

To identify protein function of the differentially expressed genes, gene ontology (GO) was used for further analysis. GO annotation analysis with the Database for Annotation, Visualization and Integrated Discovery (DAVID) annotation tool describes protein function with three basic categories: associated biological process (BP), molecular function (MF) and cellular components (CC). This categorization gives an overview of the interested protein functions. Moreover, pathway enrichment analysis was performed using the Kyoto Encyclopedia of Genes and Genomes (KEGG) pathway. Pathways that are up-regulated in EE mice compared to control mice were identified. Within the identified pathways, genes that are involved in metabolism, drug metabolism, and xenobiotic metabolism were sorted out for further validation (Tables 1– **3**).

**Table 1:** List of proteins found to be up-regulated proteins in OB of mice treated with EE.

**Accession number**	**Accession name**	**Protein name**	**Gene symbol**	**Fold change**	***P*-value**
Q80ZU7	BPIB3_MOUSE	BPI fold-containing family B member 3	Bpifb3	4.4	0.0001
P08074	CBR2_MOUSE	Carbonyl reductase [NADPH] 2	Cbr2	3.9	0.0001
Q5SQ27	Q5SQ27_MOUSE	MCG140354	Sec14l3	3.1	0.0001
Q8VCT4	CES1D_MOUSE	Carboxylesterase 1D	Ces1d	3.1	0.0001
P11679	K2C8_MOUSE	Keratin, type II cytoskeletal 8	Krt8	3.1	0.0001
P97872	FMO5_MOUSE	Dimethylaniline monooxygenase [N-oxide-forming] 5	Fmo5	2.9	0.0001
P33267	CP2F2_MOUSE	Cytochrome P450 2F2	Cyp2f2	2.9	0.0001
Q80X89	UD2A1_MOUSE	UDP-glucuronosyltransferase 2A1	Ugt2a1	2.8	0.0001
Q91X75	Q91X75_MOUSE	Cyp2a4 protein	Cyp2a5	2.7	0.0001
P05784	K1C18_MOUSE	Keratin, type I cytoskeletal 18	Krt18	2.7	0.0001
Q8K157	GALM_MOUSE	Aldose 1-epimerase	Galm	2.6	0.0001
Q8K1G6	Q8K1G6_MOUSE	Protein Muc5b	Muc5b	2.6	0.0001
P52430	PON1_MOUSE	Serum paraoxonase/arylesterase 1	Pon1	2.5	0.0001
Q8C6B0	Q8C6B0_MOUSE	MCG20149, isoform CRA_a	Mettl7a1	2.5	0.0001
Q8BHB9	CLIC6_MOUSE	Chloride intracellular channel protein 6	Clic6	2.5	0.0008
P40936	INMT_MOUSE	Indolethylamine N-methyltransferase	Inmt	2.3	0.0001
P15626	GSTM2_MOUSE	Glutathione S-transferase Mu 2	Gstm2	2.3	0.0001
P00329	ADH1_MOUSE	Alcohol dehydrogenase 1	Adh1	2.3	0.0001
P00186	CP1A2_MOUSE	Cytochrome P450 1A2	Cyp1a2	2.3	0.0001
Q9WV19	Q9WV19_MOUSE	Cytochrome P450, family 2, subfamily g, polypeptide 1	Cyp2g1	2.2	0.0001
A2BGH0	BPIB4_MOUSE	BPI fold-containing family B member 4	Bpifb4	2.1	0.0001
Q91WU0	CES1F_MOUSE	Carboxylesterase 1F	Ces1f	2.1	0.0001
O88312	AGR2_MOUSE	Anterior gradient protein 2 homolog	Agr2	2.1	0.0001
E9QN99	E9QN99_MOUSE	Alpha/beta hydrolase domain-containing protein 14B	Abhd14b	2.1	0.0001
P12265	BGLR_MOUSE	Beta-glucuronidase	Gusb	2.1	0.0001
Q9EQF5	DPYS_MOUSE	Dihydropyrimidinase	Dpys	2.1	0.0023
P30115	GSTA3_MOUSE	Glutathione S-transferase A3	Gsta3	2.1	0.0001
Q9D379	HYEP_MOUSE	Epoxide hydrolase 1	Ephx1	2.0	0.0001
Q9R092	H17B6_MOUSE	17-beta-hydroxysteroid dehydrogenase type 6	Hsd17b6	2.0	0.0015
Q9D8W7	OCAD2_MOUSE	OCIA domain-containing protein 2	Ociad2	1.9	0.0001
J3QMN6	J3QMN6_MOUSE	Dimethylaniline monooxygenase [N-oxide-forming]	Fmo6	1.9	0.0001
P56395	CYB5_MOUSE	Cytochrome b5	Cyb5a	1.9	0.0001
Q99KP3	CRYL1_MOUSE	Lambda-crystallin homolog	Cryl1	1.8	0.0001
Q5SGK3	AOXB_MOUSE	Aldehyde oxidase 3	Aox3l1	1.8	0.0001
O70475	UGDH_MOUSE	UDP-glucose 6-dehydrogenase	Ugdh	1.7	0.0001
Q7TNG8	LDHD_MOUSE	Probable D-lactate dehydrogenase, mitochondrial	Ldhd	1.7	0.0001
B2RX12	MRP3_MOUSE	Isoform 2 of Canalicular multispecific organic anion transporter 2	Abcc3	1.7	0.0011
O35945	AL1A7_MOUSE	Aldehyde dehydrogenase, cytosolic 1	Aldh1a7	1.7	0.0001
Q5SXG7	VMO1_MOUSE	Vitelline membrane outer layer protein 1 homolog	Vmo1	1.6	0.0022
Q91WR8	GPX6_MOUSE	Glutathione peroxidase 6	Gpx6	1.6	0.0001
Q9JII6	AK1A1_MOUSE	Alcohol dehydrogenase [NADP(+)]	Akr1a1	1.6	0.0001
P52196	THTR_MOUSE	Thiosulfate sulfurtransferase	Tst	1.6	0.0001
P59242	CING_MOUSE	Cingulin	Cgn	1.6	0.0014
P37040	NCPR_MOUSE	NADPH–cytochrome P450 reductase	Por	1.5	0.0001

### Molecular analysis

#### RNA extraction

Total RNA was extracted from tissue samples as previously described (30). Agilent 2100 Bioanalyzer (Agilent Technologies, Palo Alto, CA, USA) with the A260/A280 ratio for quality control analysis was used to evaluate purity of RNA extracted from olfactory bulb tissues, and visual valuation of the 28S:18S rRNA ratio using gel electrophoresis was used to evaluate the RNA integrity.

#### Quantitative real-time PCR (qRT-PCR)

To further validate proteomic analysis, qRT-PCR for interested differentially expressed genes was performed in triplicate. One microgram of purified total RNA was used for reverse transcription into cDNA using a ReverTra Ace® qPCR TR master Mix with gDNA remover (TOYOBO). The expression of each gene of interest was profiled using a LightCycler 480 (Roche Applied Science, Mannheim, Germany) using the LightCycler 480 SYBR Green master mix (Roche Applied Science). The validated genes are as follows: subfamily A7 (ALDH1A7); Carboxylesterase 1D (CES1D); Cytochrome P450, family 2, subfamily a, polypeptide 5 (CYP2A5); Serum paraoxonase 1 (PON1); Cytochrome P450, family 1, subfamily a, polypeptide 2 (CYP1A2); Dihydropyrimidinase (DPYS); Alcohol dehydrogenase 1 (ADH1); Alcohol dehydrogenase [NADP(+)] (AKR1A1); Crystalline, lambda1 (CRYL1), forward 5′-3′ and reverse 5′-3′; UDP-glucuronosyltransferase 2 family, polypeptide A1 (UGT2A1); Glutathione S-transferase, alpha3 (GSTA3); Glutathione S-transferase Mu 2 (GSTM2); Epoxide hydrolase 1 (EPHX1); Thiosulfate sulfurtransferase (TST). To confirm detoxifying activity by EE, we also identified iNOS level. And glyceraldehyde-3-phosphate dehydrogenase (GADPH) was used as the loading control. The expression level of each gene of interest was obtained using the 2^−Δ*ΔCt*^ method (31). Target-gene expression was normalized relative to the expression of GAPDH and represented as fold change relative to the control. The primer sequences are described in Table S1.

#### Glutathione activity assay (ELISA)

The concentration of total glutathione, oxidized glutathione, ratio of reduced glutathione to oxidized glutathione were measured in OB extracts using the glutathione detection kit from Enzo Life Sciences (Enzo Life Sciences, East Farmingdale, NY, USA, ADI-900-160) following the manufacturer's instructions. The values were obtained at 1:40 dilution and expressed as pmol/well and each well contained the total volume of 200 μl.

#### Western blot

To measure the expression of apoptotic neuronal cells, the protein was extracted as described above. The equal amounts of protein samples (40 μg) were denatured and separated by 4–12% Bis-Tris gels in 1× NuPage MES SDS Running Buffer (Invitrogen, Eugene, OR, USA). Proteins were transferred at 4°C onto a polyvinylidene difluoride membrane (Invitrogen) in NuPage Transfer Buffer (Invitrogen) with 20% (vol/vol) methanol. Membranes were blocked and then incubated overnight at 4°C with the following antibodies: Anti-Bax (1: 1,000 dilution, Santa Cruz Biotechnology), Anti-Bcl-2 (1:1,000 dilution, Santa Cruz Biotechnology), and Anti-Actin (1:1,000 dilution, Santa Cruz Biotechnology). The next day, blots were washed three times with TBS plus Tween 20 and incubated at room temperature for 1 hr with horseradish peroxidase-conjugated secondary antibodies (1:3,000 dilution, Santa Cruz). An enhanced chemiluminescence detection system (Amersham Pharmacia Biotech, Little Chalfont, UK) was used to visualize the protein. The intensity of the protein bands was recorded with a Fujifilm LAS-3000 imager.

### Histological analysis

#### Immunohistochemistry

Immunohistochemistry was performed as previously described (32). The animals were sacrificed and perfused with 4% paraformaldehyde. The harvested brain tissues were cryosectioned at 16-μm thickness along the sagittal plane, and immunohistochemistry staining was performed on four sections over a range of >128 μm. Sections were stained with primary antibodies against Ki67 (1:200; Abcam), and secondary antibodies such as Alexa Fluor 488 goat anti-Rabbit (1:400; Invitrogen) and mounted on glass slides with fluorescent mounting medium containing 4′,6-diamidino-2-phenylindole (Vectorshield, Vector, Burlingame, CA). The stained sections were analyzed using confocal microscopy (LSM700; Zeiss, Gottingen, Germany). Ki67^+^ cell area (μm^2^) with respect to DAPI area (/μm^2^) was evaluated using ZEN Imaging Software (Blue edition; Zeiss).

#### Terminal deoxynucleotidyl transferase dUTP nick end labeling (TUNEL) assay

For analysis of apoptosis, the selected areas of the RMS were analyzed for each group. Fluorometric TUNEL assay **(**Promega, Madison, WI, USA) was conducted according to the manufacturer's protocol. Images of apoptotic cells were taken using a fluorescent microscopy (LSM700), and positive apoptotic cells (μm^2^) with respect to DAPI area (/μm^2^) were quantified using ZEN Imaging Software (Blue edition; Zeiss).

### Statistical analysis

Statistical analyses were performed using Statistical Package for Social Sciences (SPSS) software (IBM Corporation, Armonk, NY, USA; version 23.0). All data were expressed as median and interquartile range (25th−75th percentiles). Mann–Whitney *U*-tests were used to compare the difference between groups. Linear regression analysis was conducted to identify the relationship between xenobiotic metabolizing proteins and ROS detoxification (glutathione activity) and a univariate analysis was also used identify relationships between EE and xenobiotic metabolizing proteins and glutathione activity. A *P* < 0.05 was considered statistically significant.

## Results

### Environmental enrichment significantly increased the proteins associated with metabolic pathways and xenobiotic metabolism

After placing mice in either SC cage or EE cage for 2 months, the mice were sacrificed according to the time schedule (Figure 1D). The proteomic analysis revealed that 44 up-regulated proteins were identified in EE mice compared with control mice with a cut-off fold change >1.5 (*p* < 0.05) (Table 1). The up-regulated proteins in the OB of mice exposed to EE were categorized based on biological processes (BP), molecular functions (MF), and cellular components (CC) (Table 2). BP based GO analysis identified the up-regulated proteins were related to “oxidation-reduction process” (GO:0055114), “response to toxic substance” (GO:0009636), “epoxygenase P450 pathway” (GO:0019373), “metabolic process” (GO:0008152), “short-chain fatty acid catabolic process” (GO:0019626), “aromatic compound catabolic process” (GO:0019439), “ethanol catabolic process” (GO:0006068), “cellular aromatic compound metabolic process” (GO:0006725), “cellular response to cadmium ion” (GO:0071276), “retinoic acid metabolic process” (GO:0042573), and “drug metabolic process” (GO:0017144). Moreover, in Kyoto Encyclopedia of Genes and Genomes (KEGG) pathway, the most highlighted cluster in the up-regulated genes contains overall themes for metabolic pathways and xenobiotic metabolism (Table 3).

**Table 2:** Gene ontology with Database for Annotation, Visualization, and Integrated Discovery (DAVID) generated by EE up-regulated genes.

**Category**	**ID**	**Term**	**Count**	**%**	***P*-value**	**Genes**
GOTERM_BP_DIRECT	GO:0055114	Oxidation-reduction process	15	34.88	<0.0001	CYP2F2, LDHD, UGDH, CYB5A, CYP1A, POR, FMO5, CRYL1, FMO6, CBR2, AKR1A1, GPX6, ADH1, HSD17B6, ALDH1A7
GOTERM_BP_DIRECT	GO:0009636	Response to toxic substance	5	11.63	<0.0001	CYP2F2, PON1, EPHX1, CES1D, INMT
GOTERM_BP_DIRECT	GO:0019373	Epoxygenase P450 pathway	3	6.98	0.0019	CYP2G1, CYP2F2, CYP2A5
GOTERM_BP_DIRECT	GO:0008152	Metabolic process	6	13.95	0.0030	GSTM2, GSTA3, GUSB, UGDH, UGT2A1, ALDH1A7
GOTERM_BP_DIRECT	GO:0019626	Short-chain fatty acid catabolic process	2	4.65	0.0043	CES1F, CES1D
GOTERM_BP_DIRECT	GO:0019439	Aromatic compound catabolic process	2	4.65	0.0129	PON1, EPHX1
GOTERM_BP_DIRECT	GO:0006068	Ethanol catabolic process	2	4.65	0.0150	ADH1, ALDH1A7
GOTERM_BP_DIRECT	GO:0006725	Cellular aromatic compound metabolic process	2	4.65	0.0150	EPHX1, CYP1A2
GOTERM_BP_DIRECT	GO:0071276	Cellular response to cadmium ion	2	4.65	0.0256	CYP2A5, CYP1A2
GOTERM_BP_DIRECT	GO:0042573	Retinoic acid metabolic process	2	4.65	0.0319	ADH1, ALDH1A7
GOTERM_BP_DIRECT	GO:0017144	Drug metabolic process	2	4.65	0.0381	FMO5, CYP1A2
GOTERM_MF_DIRECT	GO:0016491	Oxidoreductase activity	13	30.23	<0.0001	FMO5, CRYL1, CBR2, GPX6, CYP2F2, AKR1A1, ADH1, LDHD, UGDH, HSD17B6, ALDH1A7, CYP1A2, POR
GOTERM_MF_DIRECT	GO:0016712	Oxidoreductase activity, acting on paired donors, with incorporation or reduction of molecular oxygen, reduced flavin or flavoprotein as one donor, and incorporation of one atom of oxygen	4	9.30	0.0001	CYP2G1, CYP2F2, CYP2A5, CYP1A2
GOTERM_MF_DIRECT	GO:0050660	Flavin adenine dinucleotide binding	4	9.30	0.0006	FMO5, FMO6, LDHD, POR
GOTERM_MF_DIRECT	GO:0020037	Heme binding	5	11.63	0.0006	CYP2G1, CYP2F2, CYP2A5, CYB5A, CYP1A2
GOTERM_MF_DIRECT	GO:0019899	Enzyme binding	6	13.95	0.0016	GSTM2, CYP2A5, EPHX1, CYB5A, CYP1A2, POR
GOTERM_MF_DIRECT	GO:0004497	Monooxygenase activity	4	9.30	0.0018	FMO5, FMO6, CYP2F2, CYP1A2
GOTERM_MF_DIRECT	GO:0050661	NADP binding	3	6.98	0.0034	FMO5, FMO6, POR
GOTERM_MF_DIRECT	GO:0008392	Arachidonic acid epoxygenase activity	3	6.98	0.0049	CYP2G1, CYP2F2, CYP2A5
GOTERM_MF_DIRECT	GO:0008395	Steroid hydroxylase activity	3	6.98	0.0065	CYP2G1, CYP2F2, CYP2A5
GOTERM_MF_DIRECT	GO:0042803	Protein homodimerization activity	7	16.28	0.0081	GSTM2, CRYL1, GALM, ADH1, CLIC6, PON1, AGR2
GOTERM_MF_DIRECT	GO:0005506	Iron ion binding	4	9.30	0.0117	CYP2G1, CYP2F2, CYP2A5, CYP1A2
GOTERM_MF_DIRECT	GO:0004499	N,N-dimethylaniline monooxygenase activity	2	4.65	0.0199	FMO5, FMO6
GOTERM_MF_DIRECT	GO:0016705	Oxidoreductase activity, acting on paired donors, with incorporation or reduction of molecular oxygen	3	6.98	0.0206	CYP2F2, CYP2A5, CYP1A2
GOTERM_MF_DIRECT	GO:0004745	Retinol dehydrogenase activity	2	4.65	0.0459	ADH1, HSD17B6
GOTERM_CC_DIRECT	GO:0031090	Organelle membrane	7	16.28	<0.0001	FMO5, FMO6, CYP2F2, CYP2A5, EPHX1, CYB5A, CYP1A2
GOTERM_CC_DIRECT	GO:0070062	Extracellular exosome	18	41.86	<0.0001	GSTA3, GUSB, UGDH, DPYS, ABHD14B, CYB5A, SEC14L3, TST, CRYL1, GALM, KRT18, AKR1A1, KRT8, CLIC6, PON1, METTL7A1, VMO1, MUC5B
GOTERM_CC_DIRECT	GO:0043231	Intracellular membrane-bounded organelle	10	23.26	<0.0001	FMO5, CYP2F2, GUSB, PON1, HSD17B6, EPHX1, CYB5A, CYP1A2, POR, MUC5B
GOTERM_CC_DIRECT	GO:0005783	Endoplasmic reticulum	11	25.58	0.0004	FMO5, CYP2F2, GUSB, CES1F, HSD17B6, EPHX1, CYB5A, CES1D, CYP1A2, AGR2, POR
GOTERM_CC_DIRECT	GO:0005789	Endoplasmic reticulum membrane	8	18.60	0.0007	FMO5, FMO6, CYP2F2, CYP2A5, EPHX1, CYB5A, CYP1A2, POR
GOTERM_CC_DIRECT	GO:0005829	Cytosol	10	23.26	0.0116	GSTM2, CRYL1, AKR1A1, ADH1, UGDH, CES1F, DPYS, CES1D, ABHD14B, INMT
GOTERM_CC_DIRECT	GO:0005615	Extracellular space	8	18.60	0.0385	TST, AKR1A1, GUSB, CES1F, PON1, CES1D, AGR2, MUC5B

**Table 3:** Kyoto Encyclopedia of Genes and Genomes (KEGG) pathways identified to be significantly up-regulated from EE using Database for Annotation, Visualization, and Integrated Discovery (DAVID) software.

**Category**	**Term**	**Count**	**%**	***P*-value**	**Genes**
KEGG_PATHWAY	Metabolic pathways	16	37.20930233	<0.0001	GUSB, UGDH, DPYS, CYP1A2, TST, CRYL1, GALM, CBR2, AKR1A1, ADH1, CYP2A5, PON1, HSD17B6, UGT2A1, CES1D, ALDH1A7
KEGG_PATHWAY	Metabolism of xenobiotics by cytochrome P450	8	18.60465116	<0.0001	GSTM2, GSTA3, CBR2, CYP2F2, ADH1, EPHX1, UGT2A1, CYP1A2
KEGG_PATHWAY	Drug metabolism—cytochrome P450	7	16.27906977	<0.0001	GSTM2, FMO5, GSTA3, FMO6, ADH1, UGT2A1, CYP1A2
KEGG_PATHWAY	Pentose and glucuronate interconversions	5	11.62790698	<0.0001	CRYL1, AKR1A1, GUSB, UGDH, UGT2A1
KEGG_PATHWAY	Retinol metabolism	6	13.95348837	<0.0001	ADH1, CYP2A5, HSD17B6, UGT2A1, ALDH1A7, CYP1A2
KEGG_PATHWAY	Drug metabolism—other enzymes	4	9.302325581	0.0007	GUSB, DPYS, UGT2A1, CES1D
KEGG_PATHWAY	Glutathione metabolism	3	6.976744186	0.0156	GSTM2, GSTA3, GPX6
KEGG_PATHWAY	Glycolysis / Gluconeogenesis	3	6.976744186	0.0220	GALM, AKR1A1, ADH1
KEGG_PATHWAY	Steroid hormone biosynthesis	3	6.976744186	0.0367	HSD17B6, UGT2A1, CYP1A2
KEGG_PATHWAY	Ascorbate and aldarate metabolism	2	4.651162791	0.0904	UGDH, UGT2A1

### Differentially expressed proteins (DEPs) were validated by qRT-PCR

**Figure 2:**
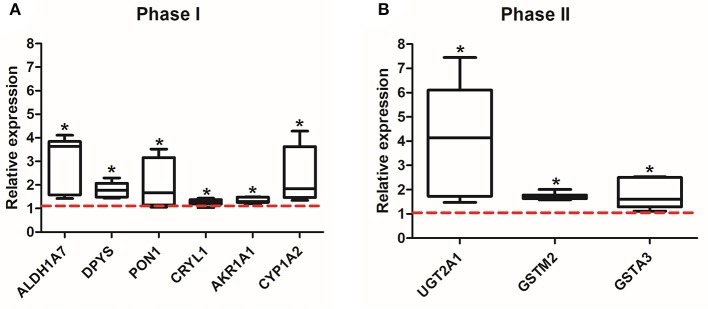
Validation of the up-regulated proteins associated with xenobiotic metabolism. Among the 44 up-regulated proteins, which were associated with xenobiotic metabolism and detoxifying enzymes, a total of 9 genes, phase I (ALDH1A7, DPYS, PON1, CRYL1, AKR1A1, CYP1A2) **(A)** and phase II (UGT2A1, GSTIM2, GSTA3) **(B)**, were significantly increased in EE mice compared to control mice (^*^p < 0.05, by Mann-Whitney U test). Values are median (interquartile range) and a red tick line indicates the relative expression of control mice.

After 8 weeks of the exposure to EE or SC, mice were sacrificed, and the olfactory bulbs were extracted and lysed for proteomic and qPCR analysis. The expression level of 44 identified proteins were significantly different in terms of the expression level between control group and EE group. The up-regulated proteins via pathway analysis were validated using qRT-PCR analysis. Xenobiotic metabolizing phase I enzymes, such as ALDH1A7, DPYS, PON1, CRYL1, AKR1A1, CYP1A2, and xenobiotic metabolizing phase II enzymes, such as UGT2A1, GSTM2, GSTA3 were significantly increased in EE mice compared with control mice (Figure 2; *p* < 0.05).

### EE enhanced glutathione activity and induced ROS detoxification

**Figure 3:**
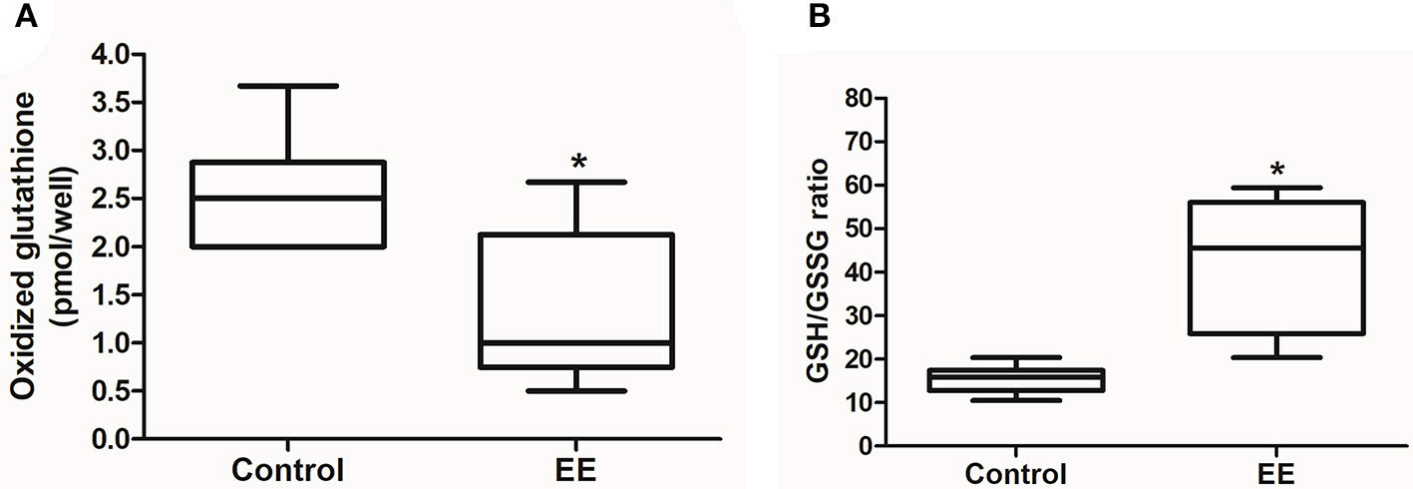
Glutathione activity. The number of oxidized glutathione in EE mice was significantly decreased compared to control mice **(A)**, and GSH/GSSG ratio was significantly increased in EE mice compared with control mice **(B)**. Values are median (interquartile range). An asterisk indicates significant difference (^*^p < 0.05, by Mann–Whitney *U*-test).

Glutathione activity is important for the removal of ROS, which generated from endogenous environment and during xenobiotic metabolism, and glutathione redox status can be a marker for oxidative stress. The number of oxidized glutathione was significantly lower in EE mice compared to control mice (Figure 3A; *p* < 0.05), and the ratio of reduced glutathione (GSH) to oxidized glutathione (GSSG) was significantly higher in EE mice compared to control mice (Figure 3B; *p* < 0.05), and no significant difference in the number of total glutathione was observed in between EE mice and control mice **(Data not shown)**.

### The significant association between xenobiotic metabolizing enzymes and glutathione activity via EE was founded

Linear regression analysis was conducted to identify which xenobiotic metabolizing enzymes mostly contribute to glutathione activity (Table 4). In the analysis of oxidized glutathione, GSTM2 (*p* = 0.008), GSTA3 (*p* = 0.022), CYP2A5 (*p* = 0.004), ALDH1A7 (*p* = 0.009), and UGT2A1 (*p* = 0.002) were found to be significantly correlated. In the analysis of GSH/GSSG, GSTM2 (*p* = 0.003), GSTA3 (*p* = 0.008), CYP2A5 (*p* = 0.029), ALDH1A7 (*p* = 0.004), UGT2A1 (*p* = 0.003), CRYL1 (*p* = 0.049), AKR1A1 (*p* = 0.024), and DPYS (*p* = 0.016) were found to be significantly correlated. The final stepwise multiple regression model revealed a significant association between oxidized glutathione and UGT2A1 (*p* = 0.002), and significant associations between GSH/GSSG and GSTM2 (*p* = 0.024) and GSH/GSSG and UGT2A1 (*p* = 0.029). EE was significantly associated with GSTM2 (Coefficient (SE) = 0.625 (0.072); reference = SC; *p* < 0.001 and UGT2A1 (Coefficient (SE) = 2.688 (0.685); *p* < 0.001), individually. Also, EE was significantly correlated with glutathione activity-related factors including oxidized glutathione [Coefficient (SE) = −1.186 (0.319); *p* = 0.002] and the ratio of GSH to GSSG [Coefficient (SE) = 26.218 (5.403); *p* < 0.001], respectively.

**Table 4:** Linear regression analysis of xenobiotic metabolism-related genes for predicting glutathione activity.

**Factor**	**Oxidized Glutathione**				**GSH/GSSG**			
**Model**	**Univariate analysis**		**Multivariate analysis**		**Univariate analysis**		**Multivariate analysis**	
**Genes**	**Coefficient (SE)**	***p*-value**	**Coefficient (SE)**	***p*-value**	**Coefficient (SE)**	***p*-value**	**Coefficient (SE)**	***p*-value**
GSTM2	−1.526 (0.505)	0.008^*^	–	–	32.762 (9.241)	0.003^*^	22.895	0.024^*^
GSTA3	−0.881 (0.346)	0.022^*^	–	–	19.249 (6.399)	0.008^*^	–	–
CYP2A5	−0.587 (0.176)	0.004^*^	–	–	9.231 (3.837)	0.029^*^	–	–
ALDH1A7	−0.432 (0.145)	0.009^*^	–	–	8.993 (2.719)	0.004^*^	–	–
UGT2A1	−0.307 (0.083)	0.002^*^	−0.307	0.002^*^	5.768 (1.678)	0.003^*^	3.916	0.029^*^
CRYL1	−1.863 (1.052)	0.096	NA		42.259 (19.818)	0.049^*^	–	–
AKR1A1	−1.333 (0.699)	0.075			32.038 (12.823)	0.024^*^	–	–
DPYS	−0.517 (0.267)	0.071			12.925 (4.817)	0.016^*^	–	–
PON1	−0.111 (0.214)	0.611			5.318 (3.994)	0.202	NA	

* *p < 0.05; SE, standard error; NA, not associated. Linear regression analysis was conducted to identify significantly xenobiotic metabolism-related genes affecting oxidized glutathione (OG) and the ratio of reduced glutathione (GSH) to oxidized glutathione (GSSG) in olfactory bulb of mouse brain. The stepwise multiple linear regressions included UGT2A1 and GSTM2 in the final models*.

### EE mediated neuroprotection and preserved newly generated cells

**Figure 4:**
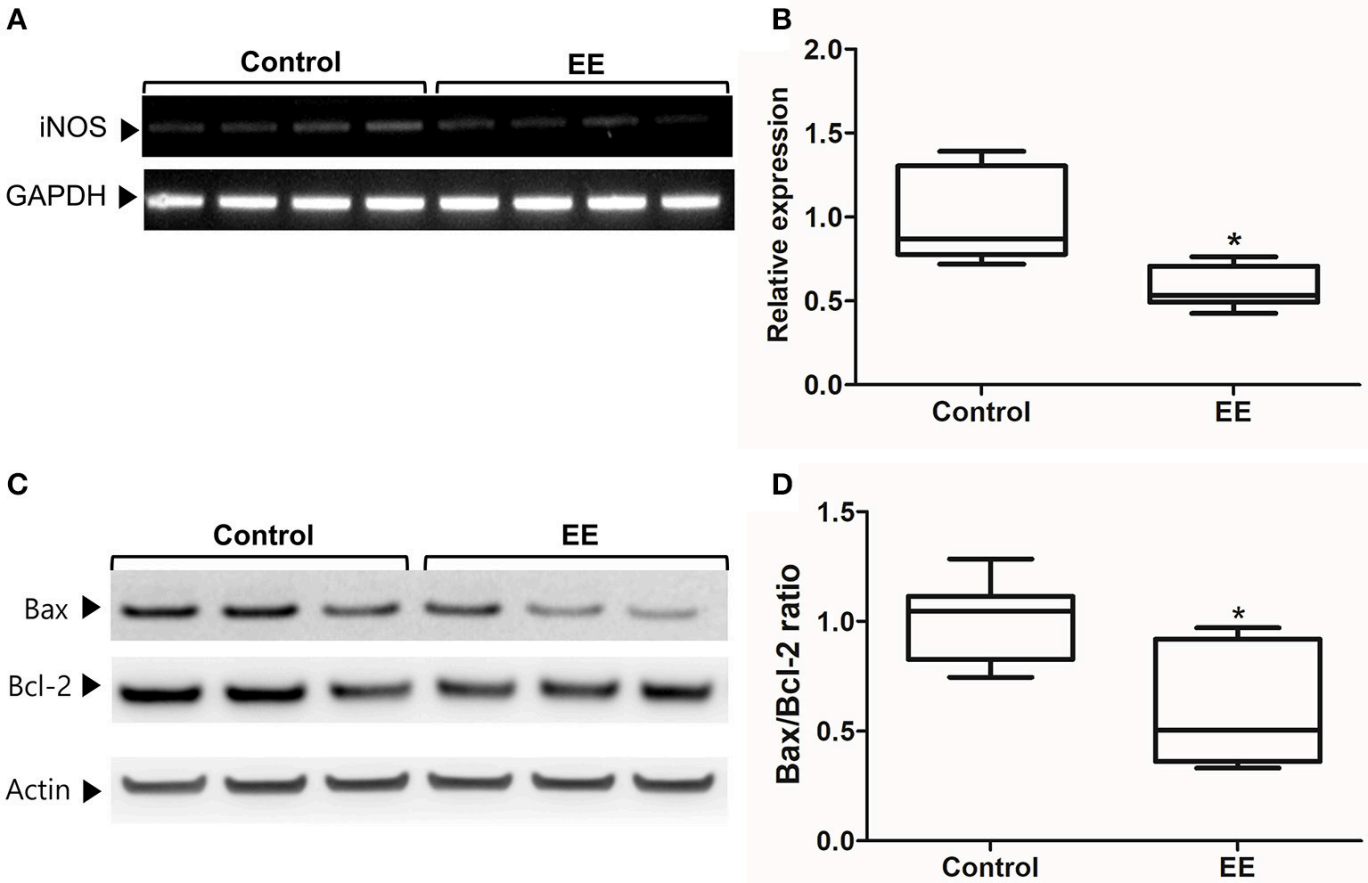
iNOS expression and apoptotic process. The expression of iNOS in the OB of EE mice were significantly decreased compared to control mice **(A,B)**. Moreover, the ratio of Bax to Bcl-2 in EE mice was significantly decreased compared to control mice **(C,D)**. Values are median (interquartile range). An asterisk indicates significant difference (^*^p < 0.05, by Mann–Whitney *U*-test).

**Figure 5:**
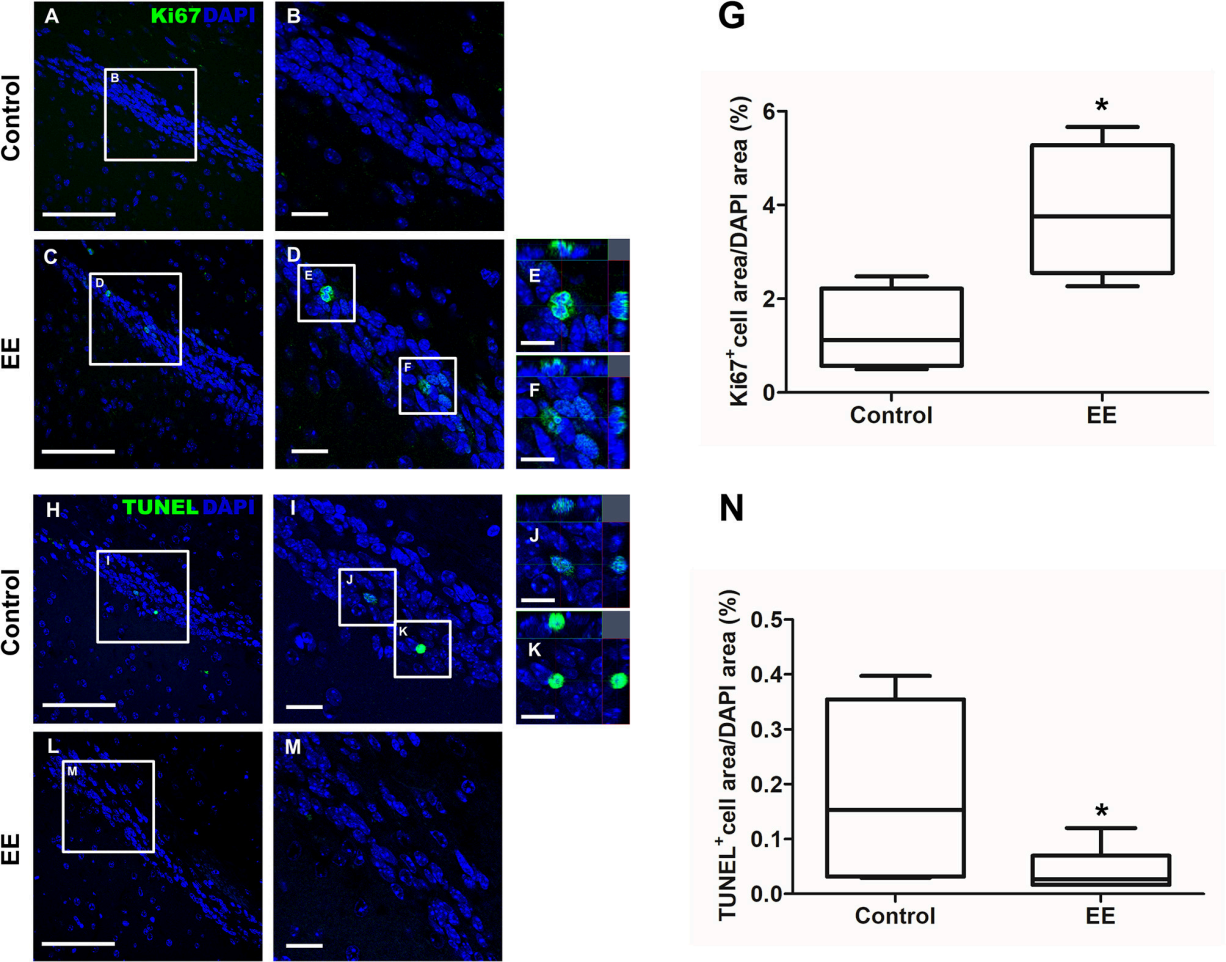
Histological assessments for neuroprotection and cell proliferation in the RMS. TUNEL^+^ cells were indicated in green **(A–F)**. TUNEL^+^ cells in EE mice were significantly decreased in the RMS compared to control mice (**G**; *p* < 0.05). Ki67^+^ cells were indicated in green **(H–M)**. Ki67^+^ cells in EE mice were significantly increased in the RMS compared to control mice (**N**; *p* < 0.05). Values are median (interquartile range). An asterisk indicates significant difference (^*^p < 0.05, by Mann–Whitney *U*-test). Scale bars for **(A,C,H,L)** represent 100 μm, scale bars for **(B,D,I,M)** represent 20 μm, and scale bars for **(E,F,J,K)** represent 10 μm.

The expression of iNOS is highly up-regulated when the cells are in oxidative stress, and the relationship between apoptotic markers (Bax and Bcl-2) is important for regulating cell apoptosis. To measure the expression of iNOS, RT-PCR was performed with the referenced primers (Table S1) (33). The representative figures of RT-PCR for the iNOS expression between EE and control group were shown in Figure 4A. The expression of iNOS in EE mice was significantly lower than that of control mice (Figure 4B; *p* < 0.05). To further evaluate the apoptotic process and neuroprotection in the OB, western blot was conducted. The representative figures of WB for Bax, Bcl-2, and Actin between EE and control group were shown in Figure 4C. The ratio of Bax, a pro-apoptosis marker to Bcl-2, an anti-apoptosis marker was significantly decreased in EE mice compared to control mice (Figure 4D; *p* < 0.05). Taken together, the upregulation of xenobiotic metabolizing enzymes mediated by EE induces glutathione-mediated ROS detoxification, eventually reducing oxidative stress and apoptosis process. IHC was performed to evaluate neuroprotection and cell proliferation in the RMS. The RMS is a structure that have a full of neuroblasts, which originated from the SVZ. These neuroblasts further migrate along the RMS into the OB, resulting in the replacement of OB interneurons. In the analysis of TUNEL assay, apoptotic cells were significantly decreased in the RMS of EE mice compared to control mice (Figures 5A–G; *p* < 0.05) In the analysis of newly generated cells, Ki67^+^ cells in the RMS were significantly increased in EE mice compared with control mice (Figures 5H–N; *p* < 0.05).

## Discussion

In this study, we provided evidences that EE has the protective effects on the OB through setting an optimal status for antioxidant and xenobiotic metabolism. The proteomic analysis revealed the DEPs in the OB of EE mice compared with control mice. GO analysis was conducted to identify protein function with DAVID 6.8 beta annotation tool and KEGG pathways. In the KEGG pathways, the most highlighted themes were general metabolism and xenobiotic metabolism within several biological processes.

To validate the expression of the up-regulated proteins, qRT-PCR was performed. Among the phase I metabolizing enzymes, ALDH1A7, DPYS, PON1, CRYL1, AKR1A1, CYP1A2 were found to be significantly increased in EE mice compared with control mice and among the phase II metabolizing enzymes, UGT2A1, GSTM2, GSTA3 were found to be significantly increased in EE mice compared with control mice. The activation ratio of phase I and phase II xenobiotic metabolism is important because the product of phase I could be more deleterious than the original form of xenobiotics. The phase-II genes encode for a battery of enzymes that are essential in the antioxidation and detoxification of xenobiotics and endogenous reactive electrophilic compounds (34). The transcriptional regulation of the phase II genes is achieved by the binding of nuclear factor erythroid2- related factor (Nrf2) to the consensus antioxidant response element (ARE) sequence in the promoter regions.

Nrf2 has been shown to regulate the expression of a group of genes encoding cytoprotective enzymes in response to cell exposure to certain toxicants, including reactive oxygen species (ROS), electrophiles, as well as dithiolethiones, isothiocyanates, and triterpenoids (35). The target genes include ones encoding such enzymes as antioxidative, catalyzing electrophile conjugation, glutathione homeostasis, producing reducing equivalents, proteasome function and other. Among others, glutathione reductase catalyzes the reduction of glutathione disulfide (GSSG) to the sulfhydryl form glutathione (GSH), which is a critical molecule in resisting oxidative stress and maintaining the reducing environment of the cell (36).

To further analyze the rate of phase II enzyme activity, one of the phase II enzymes, glutathione, the GSH/GSSG detection assay was performed. The GSH/GSSG ratio in the OB of EE mice was significantly higher than in the OB of control mice, and oxidized glutathione was significantly decreased in EE mice compared to control mice. However, the level of total glutathione was not significantly different between the groups. These results suggest that EE has beneficial effects on the OB of the mouse brain by setting an optimal status for getting rid of ROS via enhancement of antioxidant and detoxifying enzyme activity.

To further investigate the relationship between glutathione-mediated ROS detoxification and the validated up-regulated metabolizing enzymes, the regression analyses were conducted to identify whether the genes predict the glutathione activity. While GSTM2, GSTA3, CYP2A5, ALDH1A7, UGT2A1 were correlated in oxidized glutathione, GSTM2, GSTA3, CYP2A5, ALDH1A7, UGT2A1, CRYL1, AKR1A1, DPYS were correlated in GSH/GSSG ratio. The stepwise multiple linear regression indicated that UGT2A1 was the most influential gene in oxidized glutathione, and GSTM2 and UGT2A1 were the most influential genes in the GSH/GSSG ratio.

GSTM2 stabilizes reactive metabolites formed by CYPs, UGT, and ST, and the proper activity of GST isoenzymes is important for olfactory acuity (37). A fluctuation in the GST activity was observed in striatal neurons of Huntington's diseases, reflecting its protective role for oxidative damage to DNA, proteins, and lipids (38). The expression of GST activity is highly expressed in rat olfactory bulb compared to that of the cerebral cortex (37). Interestingly, the transcriptional level of Nrf2 is positively correlated to GST activities in most cases (39). GSTM2 also takes an important role in glutathione-mediated antioxidant activity and clearance of odorants (40).

UGTs, consisting of genetically variable family of enzymes, detoxify various exogenous xenobiotics from air pollutants to food and endogenous lipids (41). These enzymes involved in all three phases of xenobiotic metabolism (42) and are detectable in olfactive mucosa and olfactory bulb (43). Various isoforms of UGTs are highly expressed in the olfactory area. Their expression is highly related to the transfer rate of xenobiotics via the nasal pathway to the brain, the modulation of olfactory perception, and the detoxification of exogenous or endogenous compounds, which are important for physiological olfactory processing in the system (44).

During xenobiotic metabolism, the numerous amounts of ROS and reactive nitrogen species generate in the membrane of the cell and mitochondria. These reactive species can exert deleterious effects on DNA, protein, and membrane of the cell (44). Furthermore, excessive ROS generation or oxidative stress activates stress-mediated MAPKs, ultimately leading to cell death (37). The accumulation of endogenous ROS can be alleviated by either enzymatic or non-enzymatic antioxidants defense (45). In RT-PCR and WB analysis, the significant reductions of the iNOS expression and apoptotic process were observed in EE mice compared with control mice. This neuroprotective effect may be due to the up-regulation of glutathione activity and the reduction of ROS.

The OB relays various information from the olfactory epithelium, which has a direct contact with external environment, to the olfactory cortex (43, 46). When encountering and metabolizing xenobiotics, continuous neural regeneration and synaptic plasticity occur in sensory cell axons and mitral cells of the OB (6). Most of the progenitor cells from the subventricular zone that migrate along the migratory stream to the OB differentiate into granule cells (GC) (47). These cells are inhibitory interneurons, and their activity is extremely important to modulate mitral cell activity and to accurately shape the output from the OB to higher olfactory centers (38, 40). Notably, olfactory interneurons are continuously generated throughout adulthood and their integration in the OB circuits is regulated by sensory experience (48). Several studies have shown that exposing animals to an olfactory enrichment increases survival of adult-born neurons in the OB (49, 50). In the presence of powerful neurogenic stimulus, such as voluntary exercise, the proliferative capability of SVZ stem cells turns out to be more plastic (51). Moreover, EE increases neurogenesis in the granular cell layer (GCL) of the adult OB (52). Consistent with the previous studies, our results showed that EE significantly reduced the apoptosis of newly regenerated cells in the RMS.

Oxidative stress in brain is related with many factors such as environmental toxin, aging, and diseases. Previous studies have shown oxidative stress could be alleviated by environmental enrichment (17, 24, 53), and the dramatic effect can be shown in the aging rat brain (17, 24). EE also mediates various functional improvements via the increased expression of growth factors such as FGF-2 in the hypoxic ischemic mice (14). Moreover, EE also induces behavioral and functional improvement associated with synaptic plasticity and neurogenesis (54–57). However, the mechanisms underlying the effect of EE on xenobiotic metabolism in the OB are less clear. As indicated in the study of Yokota et al., EE during the perinatal period can significantly modulate the gene expression related to inflammation in the OB via the proper activation of xenobiotic metabolism, eventually resulting in neuroprotection (25). Consistent with the previous study, our results showed the beneficial effects of EE that can alleviate endogenous oxidative stress via the glutathione-mediated ROS detoxification in the adult mouse brain and can also mediate neuroprotection, resulting in more newly regenerated cells in the RMS and modulation of the OB circuits.

Previous studies suggested that the complexity of the environment is the key feature of EE that can provide a wide range of opportunities for stimulation in various brain regions (21, 58–60). In our study, we provided larger space with the items varying in size, shape, smell, and texture in EE. The active interaction between the mice and items available in the environment may lead to dramatic results in endogenous ROS detoxification of normal adult mice. However, since the activity response of antioxidant enzymes in the brain is probably dependent on various factors such as the duration of EE, sex, and strain of mice, further prospective studies to evaluate the effect of EE based on these factors are needed.

Since there is little information available for the effect of EE on the intact OB of normal mice, our study may provide a possible protective mechanism of EE. In the present study, the proteomic analysis revealed the DEPs, which are associated with metabolic pathways and xenobiotic metabolism, in EE mice compared with control mice. EE can induce the neuroprotective effects via the up-regulation of glutathione-associated antioxidant enzymes in the OB. EE also creates the optimal state against ROS by the up-regulation of GSH/GSSG ratio, down-regulation of iNOS expression and apoptotic process, and up-regulation of cell proliferation.

## Author contributions

JHS and SP equally contributed to this study. JHS performed most of the experiments, analyzed data, wrote the manuscript, and contributed to the study concept and design. SP performed molecular study, analyzed data, wrote the manuscript, and contributed to English editing. Y-KS conducted statistical analysis. B-GN managed animals. JWK and KPK conducted proteomic analysis and LC-MS analysis. HYL and S-RC wrote the manuscript, developed the study concept, and supervised the project. All authors read and approved the manuscript.

### Conflict of interest statement

The authors declare that the research was conducted in the absence of any commercial or financial relationships that could be construed as a potential conflict of interest.
