# Supplementary material for: The Effect of Environmental Enrichment on Glutathione-Mediated Xenobiotic Metabolism and Antioxidation in Normal Adult Mice
Source: Front Neurol. 2018 Jul 4;9:425. doi: 10.3389/fneur.2018.00425 (PMC6039562; doi:10.3389/fneur.2018.00425)
Supplement: Supplementary file 1 [file Image_1.pdf]

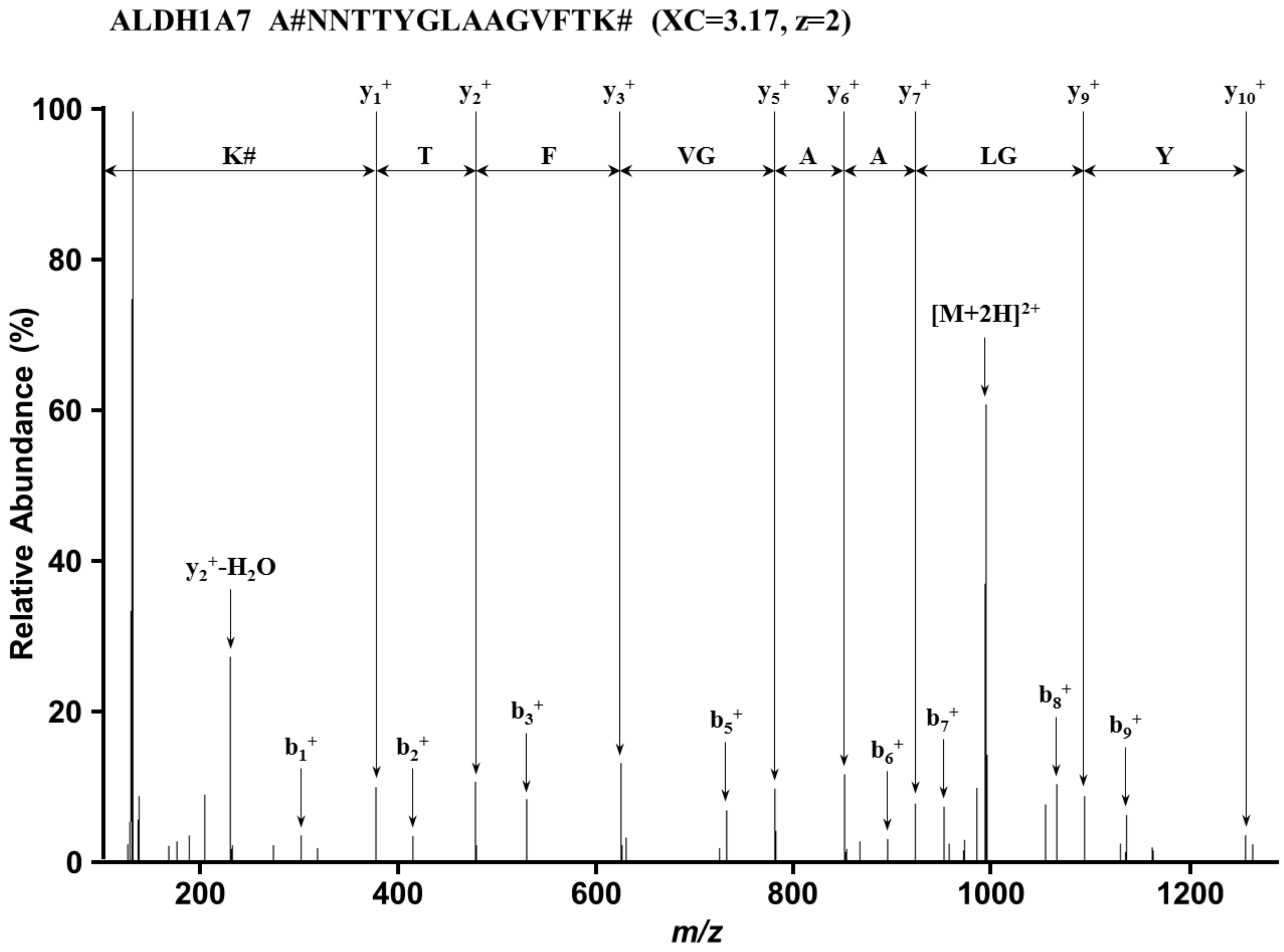

Figure S1. The mass spectrum for ALDH1A7

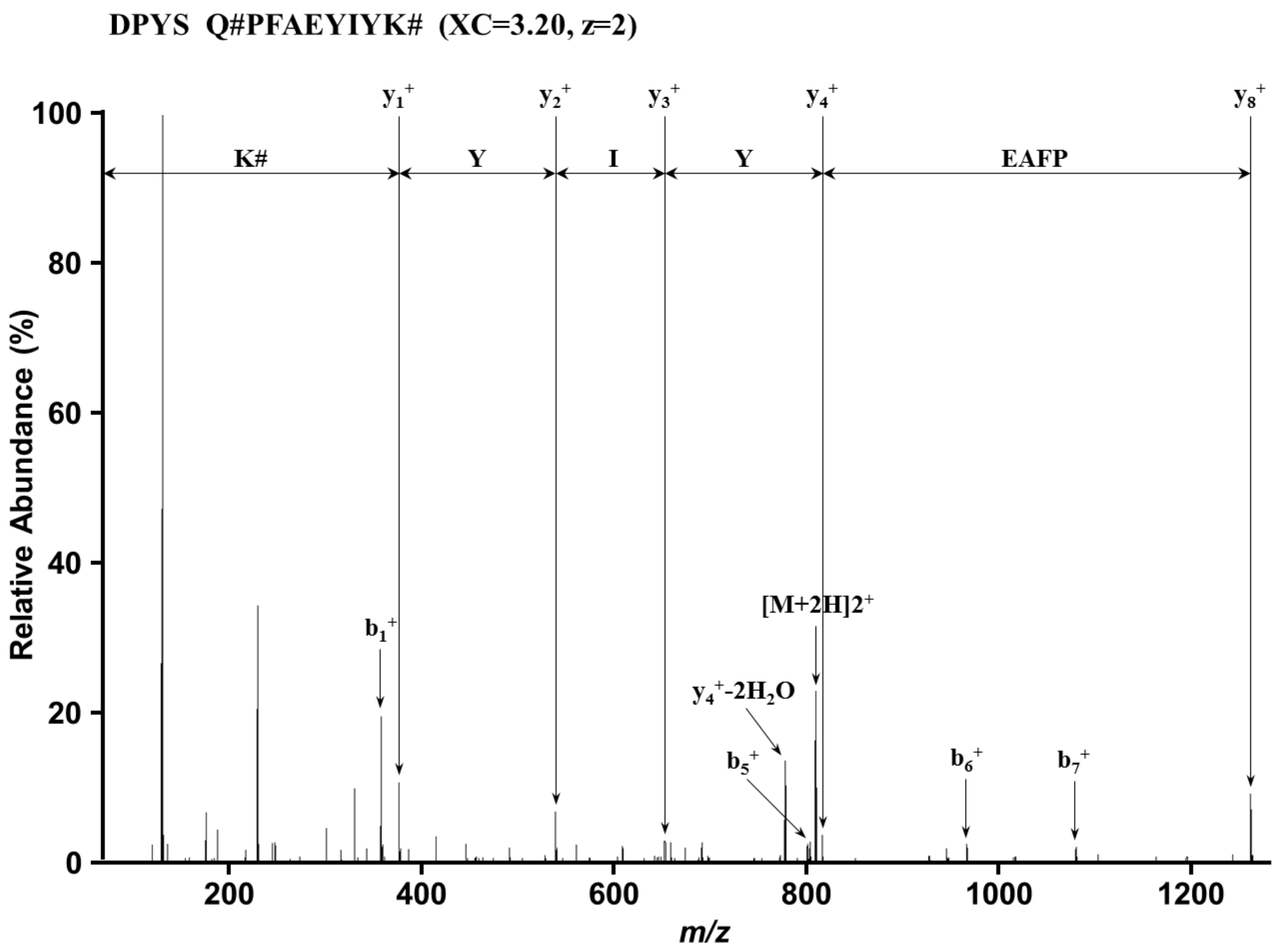

Figure S1. The mass spectrum for DPYS

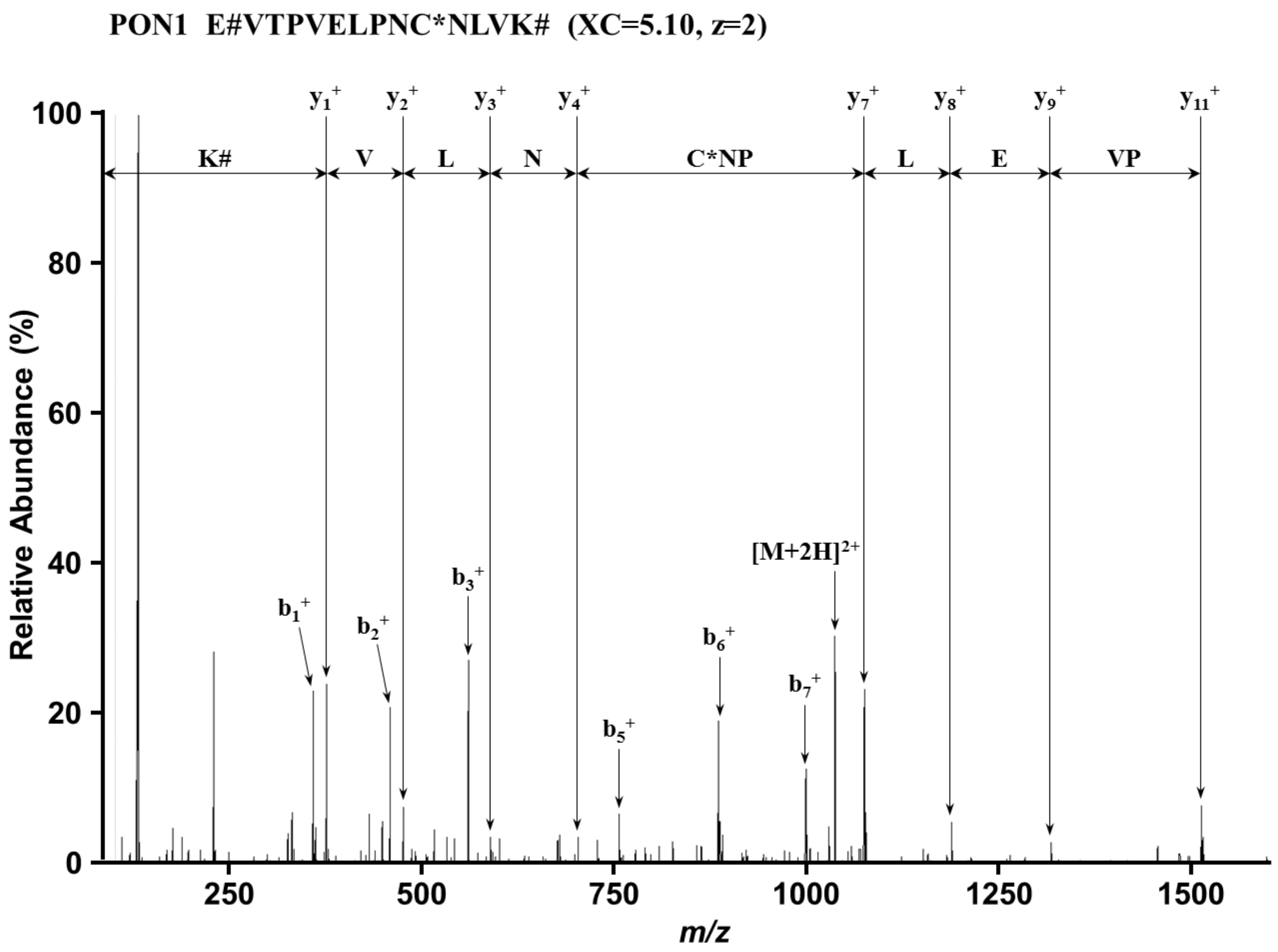

Figure S1. The mass spectrum for PON1

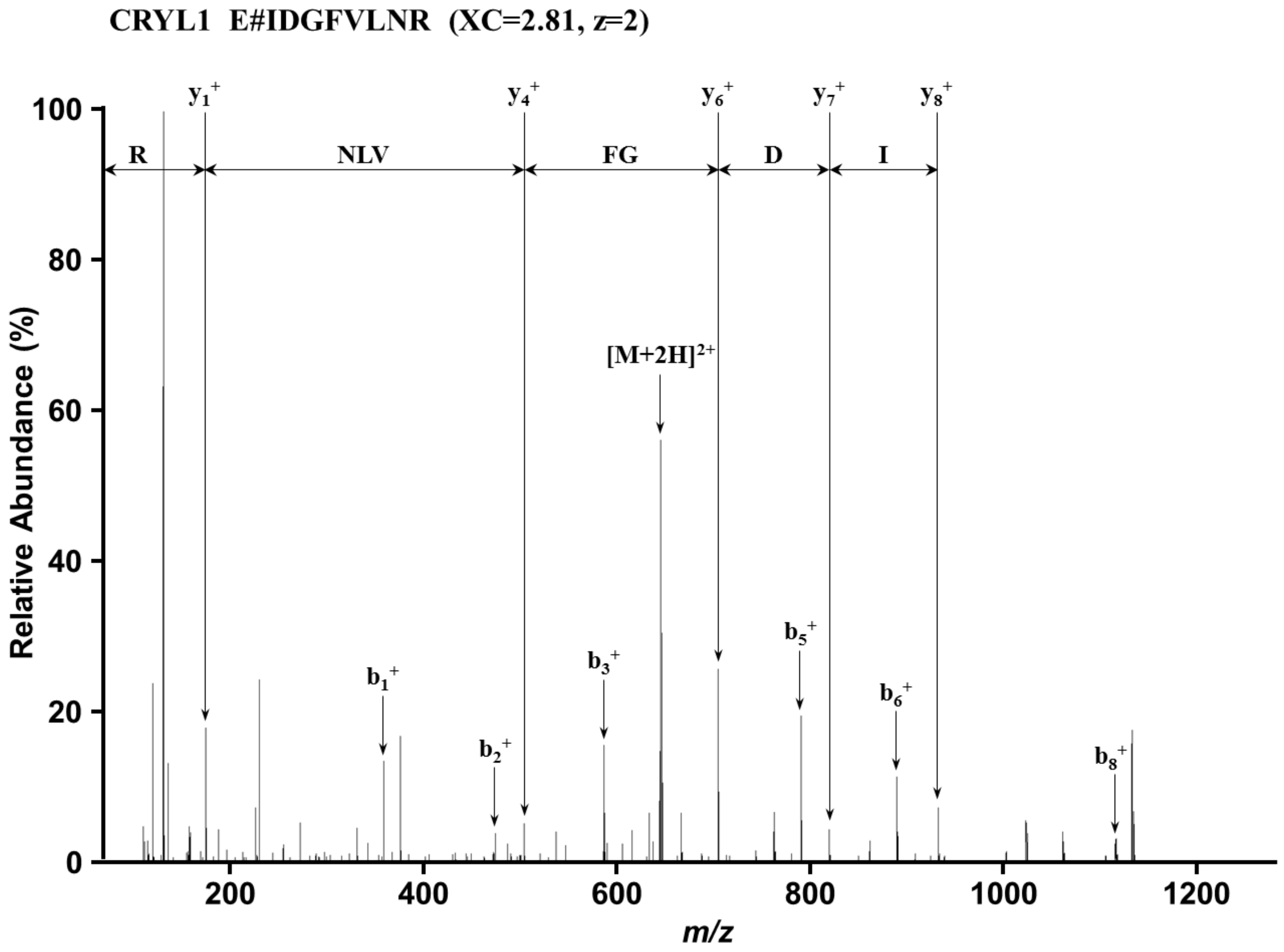

Figure S1. The mass spectrum for CRYL1

AKR1A1 H#HPEDVEPALR (XC=3.37, *z*=3)

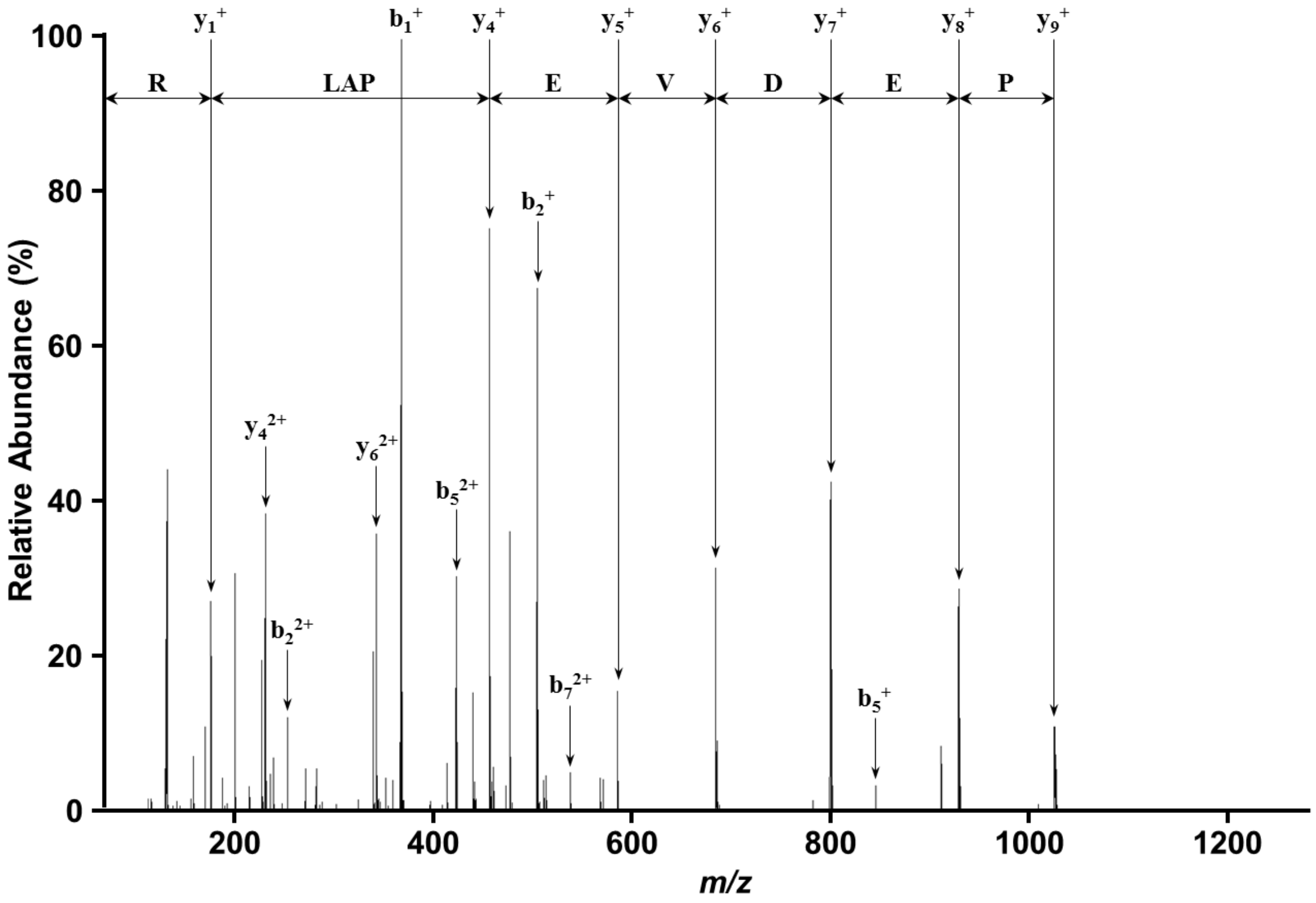

Figure S1. The mass spectrum for AKR1A1

CYP1A2 T#FNDNFVLFLQK# (XC=3.28, *z*=3)

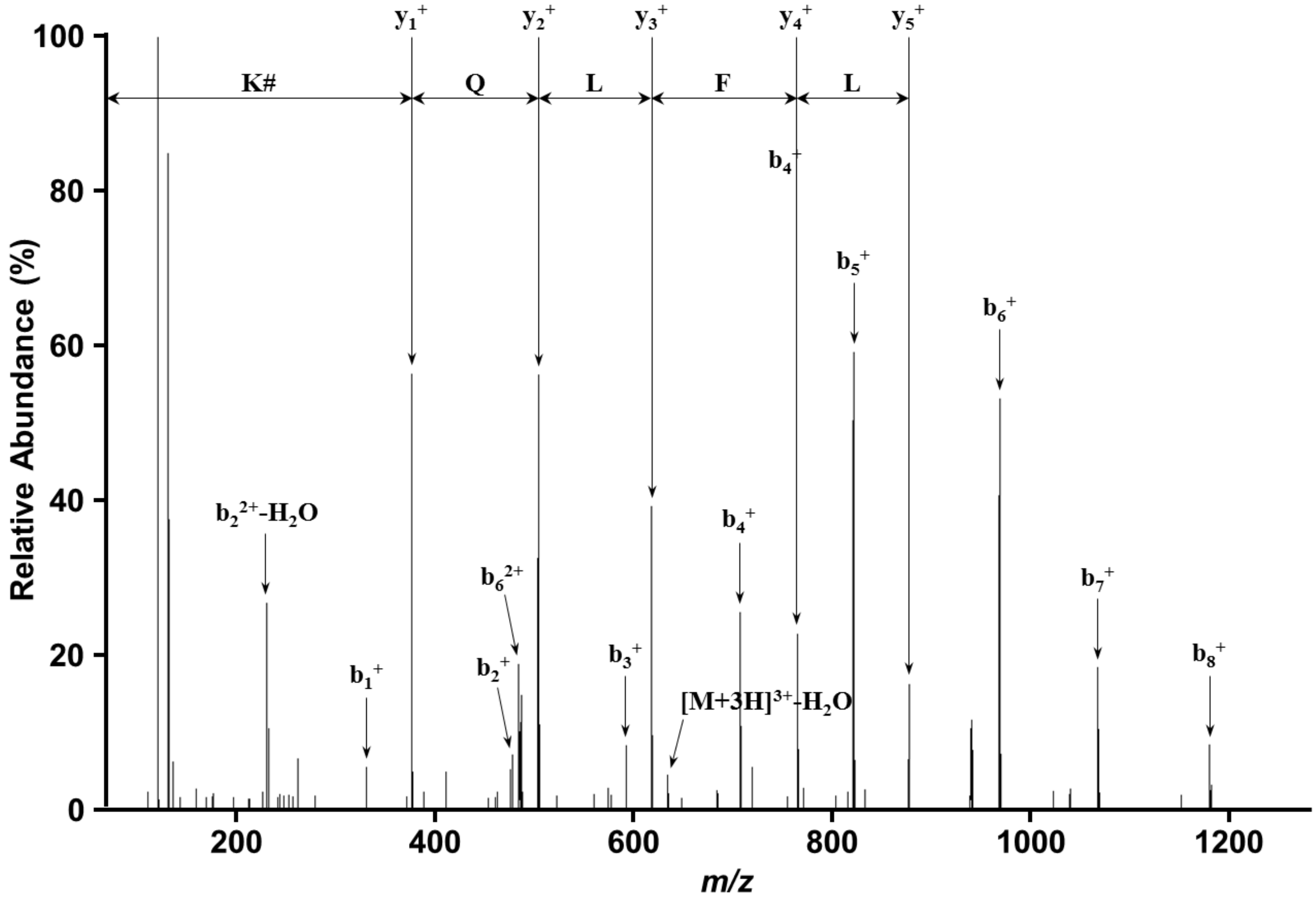

Figure S1. The mass spectrum for CYP1A2

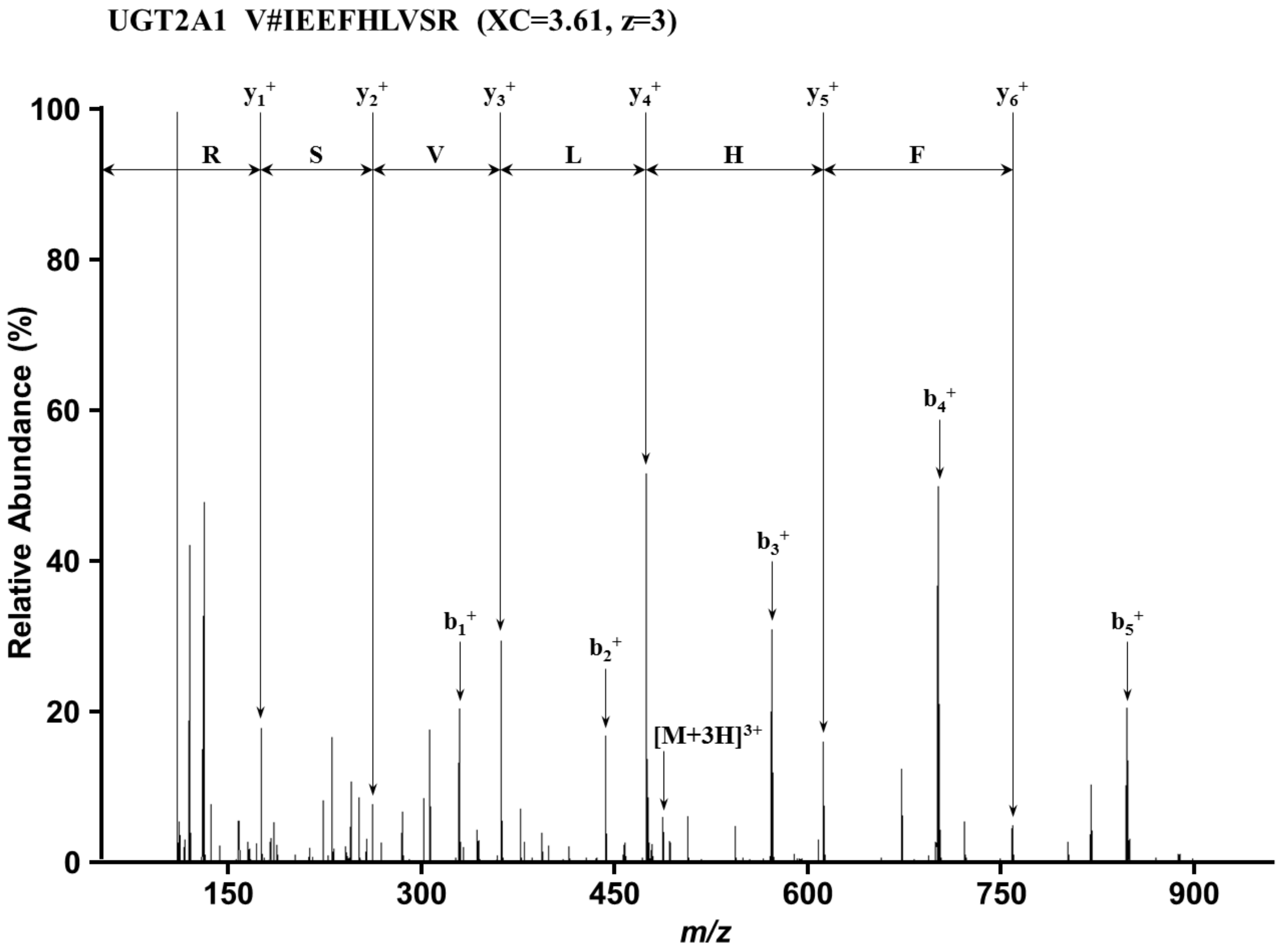

Figure S1. The mass spectrum for UGT2A1

GSTM2 Q#PWFAGNK# (XC=2.54, z=2)

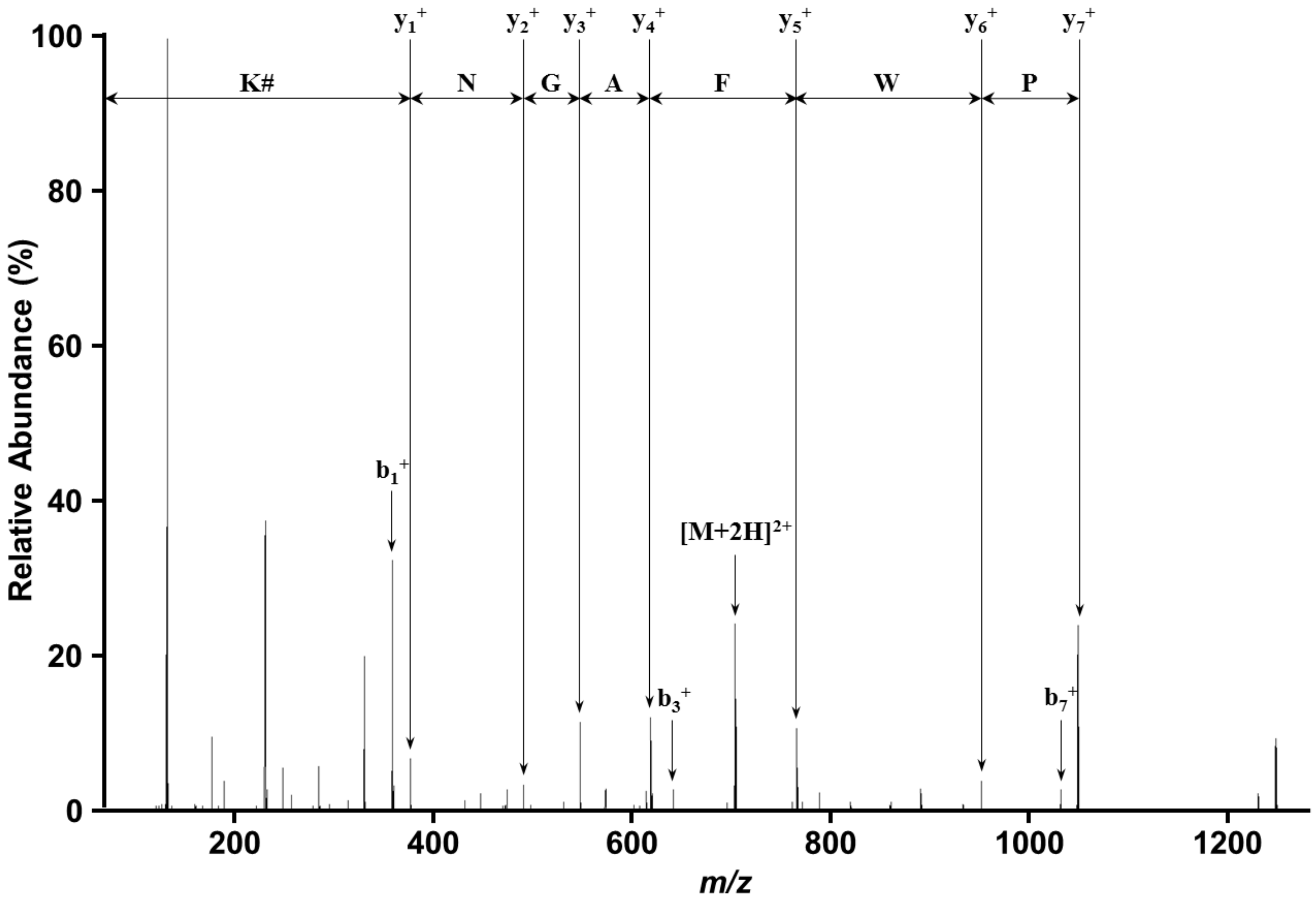

Figure S1. The mass spectrum for GSTM2

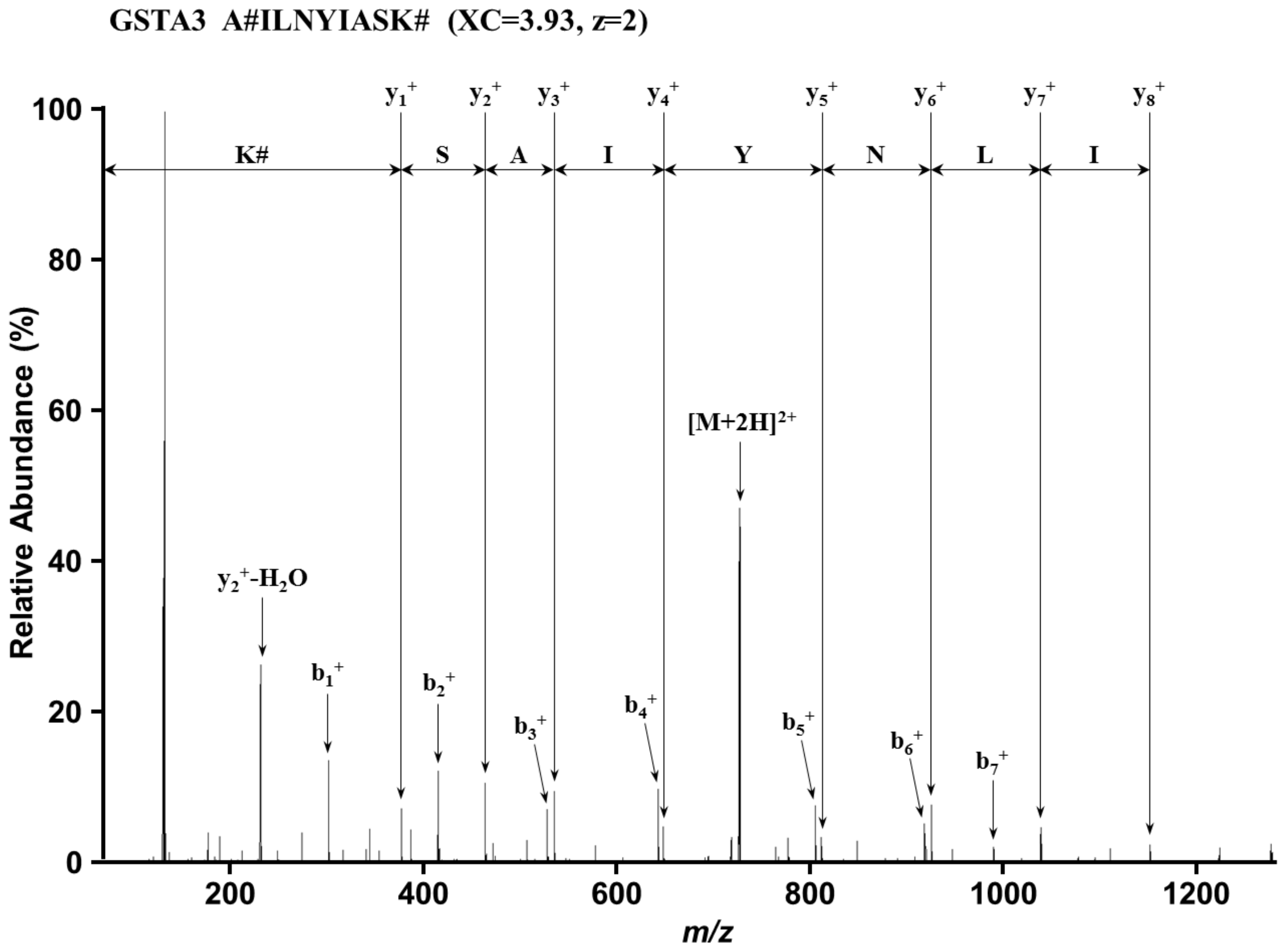

Figure S1. The mass spectrum for GSTA3
