# Supplementary material for: The Effect of Environmental Enrichment on Glutathione-Mediated Xenobiotic Metabolism and Antioxidation in Normal Adult Mice
Source: Front Neurol. 2018 Jul 4;9:425. doi: 10.3389/fneur.2018.00425 (PMC6039562; doi:10.3389/fneur.2018.00425)
Supplement: Supplementary file 2 [file Table_1.pdf]

Table S1. The sequences of primers used for qRT-PCR.

Genes	Primer sequences (5'->3')	
ALDH1A7	Forward	GGTTTAGCAGCAGGAGTCTTCA
	Reverse	CAGCCAAATAGCAGTTCACCC
PON1	Forward	ATGACGCAGAGAATCCTCCC
	Reverse	TTTGTACACAGAGGCGACCG
CYP1A2	Forward	GCTTCTCCATAGCCTCGGAC
	Reverse	TTAGCCACCGATTCCACCAC
DPYS	Forward	AGACCCGAAAGTCTGCTGTG
	Reverse	CTGAGAGAAGGCCGCATACA
AKR1A1	Forward	CCCTTTCCCAAGAATGCCGA
	Reverse	TGCCGACTGTTGAAGTTGGA
CRYL1	Forward	GATTGACGGCTTCGTCCTGA
	Reverse	GCATAGTCTCCAAGGGTCCG
UGT2A1	Forward	CTAGGAATGAGTCTTGGTGGGA
	Reverse	GGCCACAAGGACAGTCACATTA
GSTA3	Forward	TCGACGGGATGAAACTGGTG
	Reverse	TCTTTCTCCTCAGGGGGCAT
GSTM2	Forward	GAGGAGGAGAGGATTCGTGTG
	Reverse	CTTCATCTTCTCAGGGAGACCC
iNOS	Forward	TTCACCCAGTTGTGCATCGACCTA
	Reverse	TCCATGGTCACCTCCAACACAAGA
GAPDH	Forward	CAAGGTCATCCATGACAACTTTG
	Reverse	GTCCACCACCCTGTTGCTGTAG
